# Response of Hemolytic and Photosynthetic Activity of *Chattonella marina* Complex Under Variable N:P Stoichiometry

**DOI:** 10.3390/toxins18050226

**Published:** 2026-05-09

**Authors:** Xinyi Wang, Kehan Yi, Yongjun Jiang, Mengmeng Tong

**Affiliations:** 1 Food and Pharmacy Institute, Zhejiang Ocean University, Zhoushan 316022, China; wangxinyi@zjou.edu.cn (X.W.); jiangyj@zjou.edu.cn (Y.J.); 2 Ocean College, Zhejiang University, Zhoushan 316021, China; 3 Hainan Institute, Zhejiang University, Sanya 572025, China

**Keywords:** *Chattonella marina*, nitrogen limitation, phosphorus limitation, hemolytic activity, reactive oxygen species, tetrapyrrole pathway

## Abstract

*Chattonella marina* is an ichthyotoxic, bloom-forming raphidophyte known for its hemolytic activity. However, the mechanisms by which nitrogen (N) and phosphorus (P) limitation influence this hemolytic toxicity remain poorly understood. In this study, both N and P limitation reduced growth, photosynthetic efficiency (F_v_/F_m_, Y_II_, rETR_max_), and the expression of nutrient-uptake, tetrapyrrole/chlorophyll biosynthesis genes. Nevertheless, the two nutrients produced opposite effects on toxicity: N limitation lowered hemolytic activity and ROS levels to near zero, whereas P limitation kept both relatively high, similar to nutrient-replete controls. The addition of the antioxidant NAC (N-Acetyl-L-cysteine) reduced hemolytic activity, confirming that ROS contributes to toxicity. Transcriptome data showed that under N limitation, genes for nitrogen uptake and initial reduction (*NRT*, *NR*, *glnA*) were upregulated, while downstream assimilation genes (*nirA*, *GLT1*) were downregulated. In contrast, under P limitation, all the nitrogen-metabolism-related genes (*NRT*, *NR*, *glnA*, *nirA*, *GLT1*) were downregulated. In the tetrapyrrole pathway, most genes were downregulated under both nutrient-limited conditions, except for *HemD*, suggesting a bottleneck that may result in the accumulation of porphyrin intermediates within the tetrapyrrole/chlorophyll biosynthesis pathway. Together, the secondary products derived primarily from the reaction of ROS with tetrapyrrole-based compounds appear to be the main contributors to hemolytic toxicity. Consequently, high levels of both ROS and porphyrin intermediates under P-limited conditions, as well as high ROS levels but low porphyrin intermediates under nutrient-sufficient conditions, may both contribute to the high hemolytic toxicity of *C. marina*. In contrast, under N limitation, despite the accumulation of porphyrin intermediates, the strong suppression of photosynthetic electron transport limits both ROS production and the synthesis of nitrogen-containing toxins, resulting in low hemolytic activity. These findings demonstrate that nutrient conditions regulate hemolytic activity in *C. marina* in a nutrient-specific manner.

## 1. Introduction

Species within the *Chattonella marina* complex, *C. marina*, *C. antiqua* and *C. ovata* [1], hereafter collectively referred to as *C. marina* for brevity, frequently form ichthyotoxic algal blooms worldwide [2]. These blooms have caused mass mortality of fish (e.g., *Pagrus major*, *Trachurus japonicus*, *Seriola quinqueradiata*, *Thunnus maccoyii*, *Thunnus thynnus orientalis*), as well as shrimp, and shellfish in Japan [3], Mexico [4,5], Canada [6], Korea [7], India [8], New Zealand [9], Australia [10], the United States [11,12], and China [13], severely damaging coastal marine environments and aquaculture industries.

The reported bioactive compounds produced by *C. marina* include polyunsaturated fatty acids [14,15], neurotoxins [16,17], hemolytic toxins [15,18], and reactive oxygen species (ROS) [14] These compounds act collectively through direct contact, causing mortality either by suffocation [19,20,21], or by direct toxicity to gill tissues [14,22,23,24], ultimately leading to death. The synergistical effect of ROS is supported by observations of high hemolytic activity in high-ROS-producing strains of *C. marina* [25], and reduced toxicity in low-ROS strains [26]. Among these bioactive compounds, hemolytic toxins are considered the main cause of mortality and serve as a key biomarker for assessing the toxic effects of *C. marina*.

Current evidence indicates that the hemolytic activity of *C. marina* is photosensitive [18], and has the same absorbance peaks at approximately 446, 583, and 635 nm as chlorophyll *c* [18]. Further studies have confirmed that the hemolytic toxicity of *C. marina* is closely associated with Chl *a* and Chl *c* [27] and is highly sensitive to environmental factors such as light, iron concentration [27], temperature, nutrient availability [28], and salinity [29]. High hemolytic activity is detected in *C. marina* cells under light intensities above 100 µmol m^−2^ s^−1^ [27], in iron-sufficient [27] or low-salinity (below 22) environments [29]. However, the biosynthetic pathway of the hemolytic toxins in *C. marina* remains unclear.

Anthropogenic eutrophication has created extreme phosphorus limitation conditions in most coastal oceans [30,31,32,33], where *C. marina* blooms generally occur [34]. Therefore, in the present study, a series of nutrient supplements were employed as external drivers to regulate the photosynthetic and hemolytic activity of the *C. marina* complex. The physiological, molecular, and metabolic responses of *C. marina* were investigated, with particular emphasis on the variation in hemolytic activity in relation to the porphyrin synthesis pathway and the synergistical effect of ROS under relevant nutrient stress.

## 2. Results

### 2.1. Growth Response of C. marina

**Figure 1:**
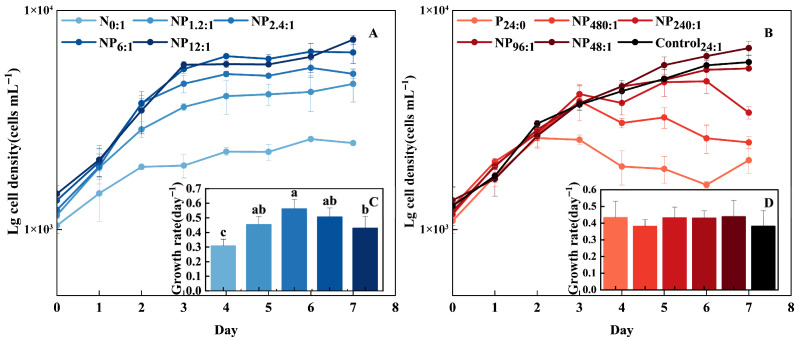
Growth curves (**A**,**B**) and growth rates (**C**,**D**) of *Chattonella marina* complex under different N:P conditions. Lowercase letters indicate significant differences among treatments (one-way ANOVA, *p* < 0.05). Data represent the mean ± SD, n = 3.

The growth rate, exponential growth period and maximum biomass of *C. marina* varied significantly with DIN concentration (Figure 1A,C). The raphidophyte *C. marina* grew exponentially for 2–5 days, reaching a maximum growth rate of 0.51 day^−1^ at DIN concentrations of 44.1, 88.2 and 221 µM (with NP ratios of 1.2:1, 2.4:1 and 6:1, respectively). Growth was significantly limited under severe DIN limitation (N_0:1_) (*p* < 0.05), where the growth rate decreased to 0.31 day^−1^. Growth was also limited under high DIN conditions (441 µM and 882 µM), with growth rates declining to 0.43 day^−1^ and 0.38 day^−1^, respectively.

In contrast, DIP primarily influenced the duration of logarithmic growth phase and the maximum biomass, but had no significant effect on the growth rate (Figure 1B, *p* > 0.05). As DIP concentration increased, the logarithmic growth phase extended from 2 to 5 days, and the maximum biomass rose from 2.07 × 10^3^ to 6.73 × 10^3^ cells mL^−1^. The growth rate remained similar across all P treatments, at approximately 0.42 day^−1^, with no significant differences.

### 2.2. Hemolytic Activity of C. marina

**Figure 2:**
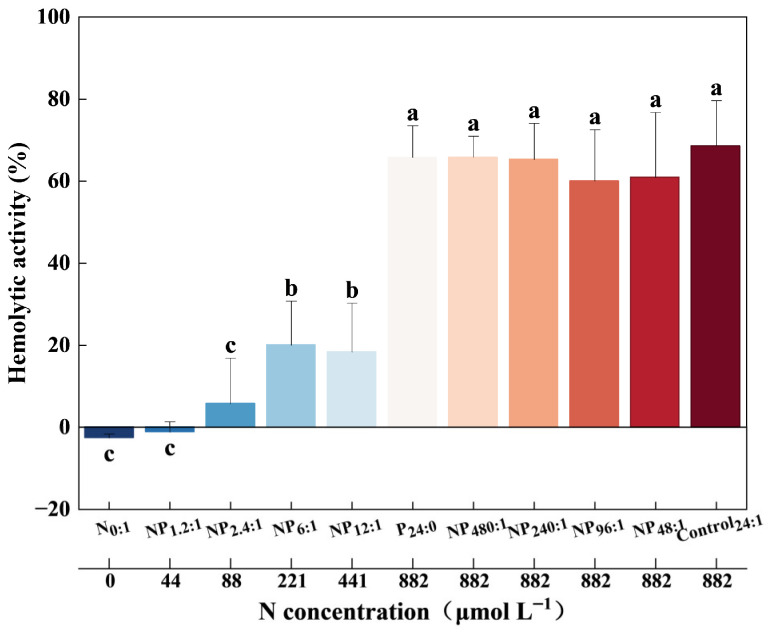
Hemolytic activity of *Chattonella marina* complex under different stoichiometry conditions. Lowercase letters indicate significant differences among treatments (one-way ANOVA, *p* < 0.05). Data represent the mean ± SD, n = 7. Hemolytic activity was normalized to 5 × 10^2^ cells mL^−1^.

Hemolytic activity in *C. marina* was significantly affected by DIN concentration but not by DIP (Figure 2). Hemolytic activity did not vary significantly with population growth over time (*p* > 0.05, Figure S2); therefore, the hemolytic activity for each treatment was represented by the average value across the sampling period (Figure 2).

Nitrogen limitation significantly suppressed the hemolytic activity of *C. marina* (Figure 2). Hemolytic activity was nearly undetectable in the N_0:1_ and NP_1.2:1_ treatments, increased to a low level of 0.5% in the NP_2.4:1_ treatment, and then rose markedly to approximately 19% in the NP_6:1_ and NP_12:1_ treatments. When DIN was sufficient (i.e., concentration is 882 µM), hemolytic activity of *C. marina* reached a maximum of 65% under both P-depleted and P-sufficient conditions.

### 2.3. Photosynthetic Activity of C. marina

**Figure 3:**
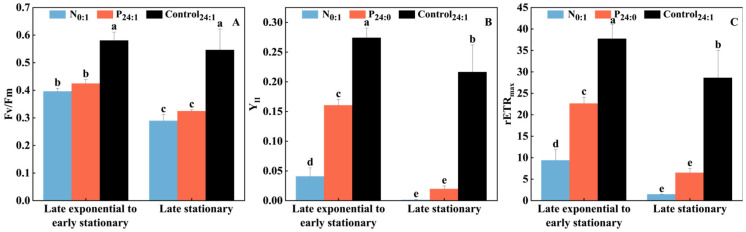
Maximum quantum yield of PSII (F_v_/F_m_, (**A**)), effective quantum yield (Y_II_, (**B**)), and the maximum relative electron transport rate (rETR_max_, (**C**)) of the *Chattonella marina* complex at the late exponential to early stationary phase (day 4~6) and late stationary phase (day 7~10) under N-limited (N_0:1_), P-limited (P_24:0_) and NP-sufficient (Control_24:1_) conditions. Lowercase letters indicate significant differences among treatments (one-way ANOVA, *p* < 0.05). Data represent the mean ± SD, n = 3.

Photosynthetic activity in *C. marina* was evaluated in N-limited (N_0:1_), P-limited (P_24:0_) and nutrient-sufficient conditions (Control_24:1_, Figure 3 and Figure S3). Both DIN and DIP limitation significantly suppressed the F_v_/F_m_ of *C. marina*, a key indicator of photosynthetic efficiency in phototrophs. The highest F_v_/F_m_ value (0.56 ± 0.02) was observed under NP-sufficient conditions, where the culture remained healthy from late exponential to late stationary phase (Figure 3A). In contrast, under NP-depleted conditions, F_v_/F_m_ declined significantly from the late exponential phase (0.41 day^−1^) to late stationary phase (0.31 day^−1^).

The effective quantum yield (Y_II_) of *C. marina*, which represents the actual efficiency of photochemical energy conversion, was also significantly inhibited under N- and P-limited conditions, particularly under N limitation (Figure 3B). Healthy cells of *C. marina* growing at the late exponential stage under nutrient-sufficient conditions exhibited the highest Y_II_ value (0.27 ± 0.02), which then declined to 0.22 ± 0.04 during the late stationary phase. Under P- and N-limited conditions, Y_II_ decreased markedly to 0.04 ± 0.01 and 0.16 ± 0.01, respectively, during late exponential growth, and declined to nearly zero in the late stationary phase (Figure 3B).

Similarly, the maximum relative electron transport rate (rETR_max_) of *C. marina*, reflecting the potential photosynthetic capacity, was significantly suppressed under N and P limitation as well, decreasing from 37.7 in healthy cells, to 22.6 under P limitation and 9.3 under N limitation during late exponential growth. The decline became more severe during the late stationary stage of *C. marina* (Figure 3C).

**Figure 4:**
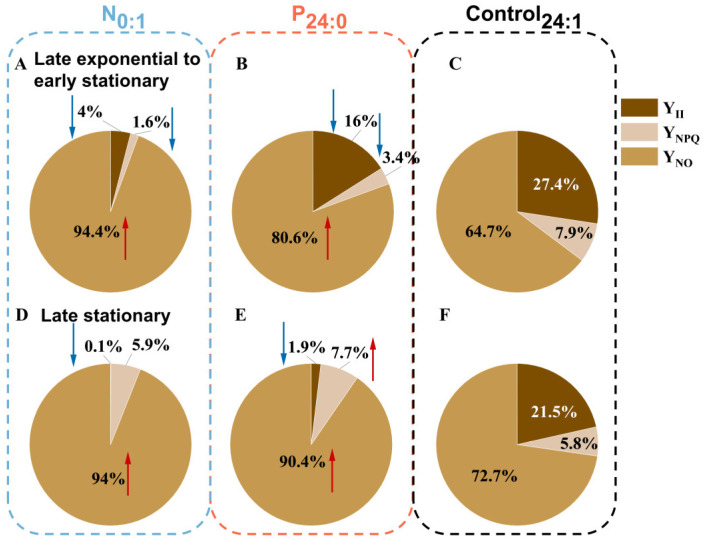
Photosynthetic energy partitioning in the *Chattonella marina* complex at the late exponential to early stationary (day 4~6) and late stationary phases (day 7~10) under N-limited (N_0:1_), P-limited (P_24:0_) and NP-sufficient (Control_24:1_) conditions. (**A**) N_0:1_, (**B**) P_24:0_, (**C**) Control_24:1_ at the late exponential to early stationary; (**D**) N_0:1_, (**E**) P_24:0_, (**F**) Control_24:1_ at the late stationary phase. Arrows indicate significant differences among treatments (one-way ANOVA, *p* < 0.05).

The light energy distribution during the growth of *C. marina* was demonstrated in Figure 4. Across all treatments, over 60% of light energy was dissipated as heat (Y_NO_). This dominance was exacerbated under nutrient limitation (Figure 4A,B) and during the late stationary phase (Figure 4D–F), particularly under N-limited conditions, where Y_NO_ rose to 94.4% and 94% during the late exponential and late stationary phase, respectively. Correspondingly, Y_II_ and Y_NPQ_ decreased significantly during the late exponential growth of *C. marina* (Figure 4A,B). In the late stationary phase, a slight but significant increase in Y_NPQ_ was observed in P-limited cells, whereas Y_II_ declined significantly across all groups (Figure 4D–F).

### 2.4. ROS Production in C. marina

**Figure 5:**
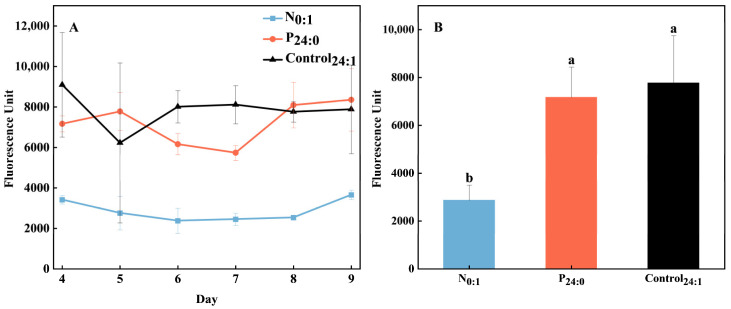
Reactive oxygen species (ROS) production during the growth (**A**) and average values (**B**) in the *Chattonella marina* complex under N-limited (N_0:1_), P-limited (P_24:0_) and NP-sufficient (Control_24:1_) conditions. Lowercase letters indicate significant differences among treatments (one-way ANOVA, *p* < 0.05). Data represent the mean ± SD, n = 3. Fluorescence units were normalized to 5 × 10^2^ cells mL^−1^.

During the growth of *C. marina*, ROS production did not vary significantly (*p* > 0.05) under P limitation or with cell growth (Figure 5A) but it declined markedly (*p* < 0.05) under N limitation (Figure 5B). ROS production in healthy *C. marina* cells was 7771 FU per 500 cells, decreasing slightly to 7166 FU per 500 cells under P limitation (*p* > 0.05) and significantly (*p* < 0.05) to 2860 FU per 500 cells under N limitation.

### 2.5. Effects of NAC on Hemolytic Activity of C. marina

**Figure 6:**
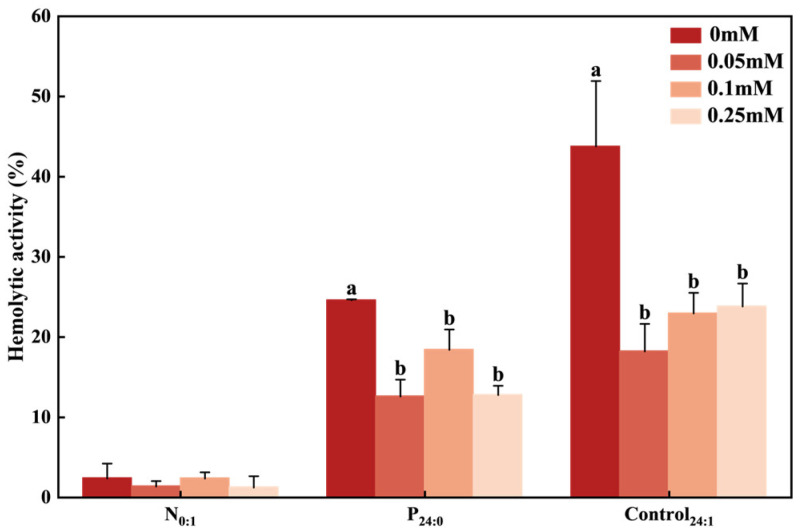
Dose-effect of N-acetyl-L-cysteine (NAC) on the hemolytic activity of *Chattonella marina* complex under N-limited (N_0:1_), P-limited (P_24:0_) and NP-sufficient (Control_24:1_) conditions. NAC was applied at final concentrations of 0, 0.05, 0.1, and 0.25 mM. Lowercase letters indicate significant differences among treatments (one-way ANOVA, *p* < 0.05). Data represent the mean ± SD, n = 3. Hemolytic activity was normalized to 5 × 10^2^ cells mL^−1^.

The addition of NAC at concentrations ≥ 0.05 mM significantly reduced the hemolytic activity of *C. marina* from 43% to 20% in the Control_24:1_ treatment, and from 24% to 15% in the P_24:0_ treatment (Figure 6). The hemolytic activity of *C. marina* in N_0:1_ was extremely low (0.02%) both in the absence and presence of NAC.

### 2.6. Gene Expression in C. marina

Transcriptomic data of *C. marina* revealed that the differentially expressed genes under N and P limitation conditions were primarily associated with photosynthesis antenna proteins, nitrogen metabolism, porphyrin and chlorophyll metabolism, glycolysis/gluconeogenesis, and fructose and mannose metabolism (Figures S4 and S5). As shown in Figure 2, hemolytic activity of *C. marina* was strongly associated with DIN concentrations and was suppressed under N limitation. By combining transcriptome analysis with the hemolytic activity response, the two most significantly enriched pathways, nitrogen metabolism and porphyrin and chlorophyll metabolism, were further analyzed.

#### 2.6.1. Nitrogen Metabolism-Related Genes Expression

**Figure 7:**
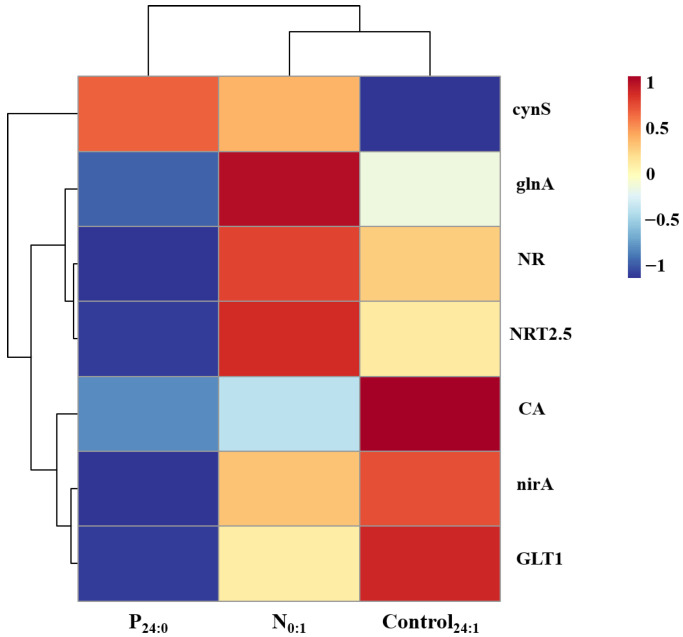
Transcript levels of nitrogen metabolism-related genes in the *Chattonella marina* complex under N-limited (N_0:1_), P-limited (P_24:0_) and NP-sufficient (Control_24:1_) conditions. Color indicates the direction of regulation: red represents up-regulation and blue represents down-regulation, with color intensity representing the magnitude of change. Data represent the mean value, n = 3.

Seven genes involved in nitrogen metabolism in *C. marina*, including *cynS*, *glnA*, *NR*, *NRT2.5*, *CA*, *nirA*, and *GLT1*, were significantly affected by nitrogen and phosphorus limitation (Figure 7). Gene expression levels were analyzed relative to the nutrient-replete control (Control_24:1_).

Under phosphorus-limited conditions (P_24:0_ vs. Control_24:1_), *cynS* was markedly upregulated by 5.3-fold, whereas *glnA*, *NR*, *NRT2.5*, *CA*, *nirA*, and *GLT1* were downregulated to 0.75-, 0.39-, 0.14-, 0.52-, 0.29-, and 0.39-fold of the control, respectively. In contrast, under nitrogen-limited conditions (N_0:1_ vs. Control_24:1_), *cynS*, *glnA*, *NR*, and *NRT2.5* were upregulated by 4.12-, 1.5-, 1.4-, and 3.24-fold, respectively, while *CA*, *nirA*, and *GLT1* were downregulated to 0.60-, 0.76-, and 0.68-fold of the control.

**Figure 8:**
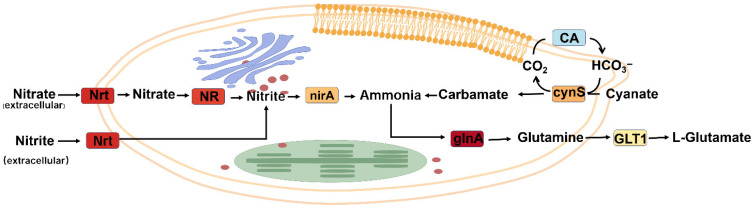
Nitrogen metabolic pathways in the *Chattonella marina* complex under nitrogen-limited conditions (N_0:1_). Color intensity corresponds to the transcript levels of nitrogen metabolism-related genes under N-limited (N_0:1_) conditions, using the same color scheme as the Figure 7.

Pathway analysis (Figure 8) revealed a coordinated regulation of nitrogen metabolism under nitrogen limitation. Genes involved in nitrogen uptake and initial reduction, including *NRT* and *NR*, were upregulated, whereas *nirA*, responsible for nitrite reduction, was downregulated. In the downstream ammonia assimilation pathway, *glnA* was upregulated, while *GLT1* was downregulated. Additionally, *CA* was downregulated, and *cynS* was upregulated. These results indicate a differential regulation of nitrogen metabolism, characterized by enhanced nitrogen uptake and initial assimilation steps coupled with reduced downstream nitrogen incorporation.

#### 2.6.2. Porphyrin and Chlorophyll Metabolism Related Genes Expression

**Figure 9:**
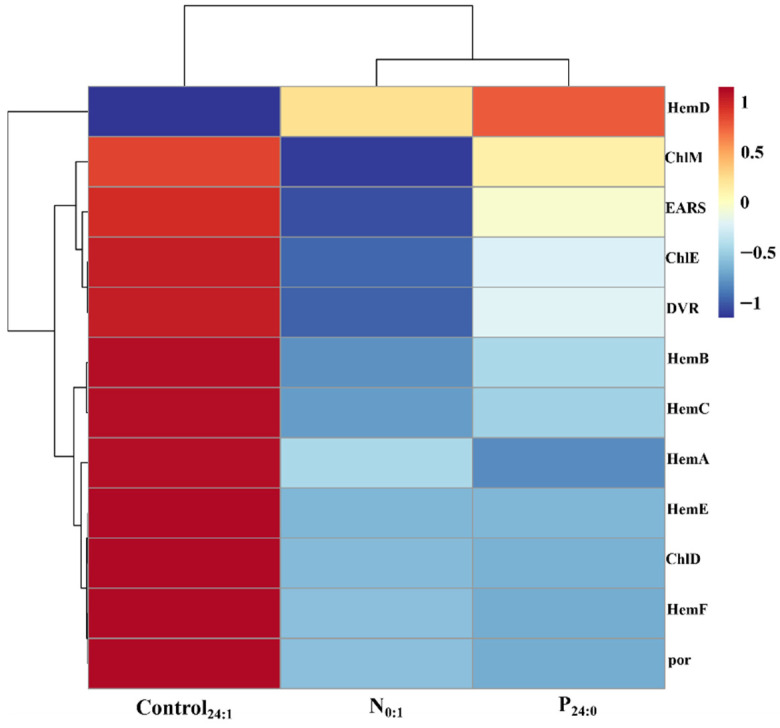
Relative transcript levels of porphyrin and chlorophyll synthesis-related genes in the *Chattonella marina* complex under N-limited (N_0:1_), P-limited (P_24:0_) and NP-sufficient (Control_24:1_) conditions. Color indicates the direction of regulation: red represents up-regulation and blue represents down-regulation, with color intensity representing the magnitude of change. Data represent the mean value, n = 3.

According to the KEGG pathway database, 45 genes are involved in the porphyrin biosynthesis pathway, and 15 genes are involved in the chlorophyll biosynthesis pathway. In this study, seven of the 45 genes (*EARS*, *HemA*, *HemB*, *HemC*, *HemD*, *HemE*, and *HemF*) and five of the 15 genes (*ChlE*, *ChlM*, *ChlD*, *DVR*, and *por*) were identified and exhibited significant expression changes (Figure 9).

In contrast to the nutrient-replete condition (Control_24:1_), all genes except *HemD* in the porphyrin and chlorophyll metabolism pathway were downregulated under N-limited and P-limited conditions. Specifically, under N-limited conditions, *EARS*, *HemA*, *HemB*, *HemC*, *HemE*, *HemF*, *ChlE*, *ChlM*, *ChlD*, *DVR*, and *por* were downregulated to 0.87-, 0.46-, 0.28-, 0.28-, 0.47-, 0.66-, 0.08-, 0.43-, 0.54-, 0.86-, and 0.74-fold of the control, respectively, whereas *HemD* was upregulated by 1.45-fold. Similarly, under P-limited conditions (P_24:0_ vs. Control_24:1_), *EARS*, *HemA*, *HemB*, *HemC*, *HemE*, *HemF*, *ChlE*, *ChlM*, *ChlD*, *DVR*, and *por* were downregulated to 0.93-, 0.39-, 0.35-, 0.33-, 0.46-, 0.64-, 0.20-, 0.73-, 0.54-, 0.91-, and 0.73-fold of the control, respectively, while *HemD* was upregulated by 1.66-fold.

**Figure 10:**
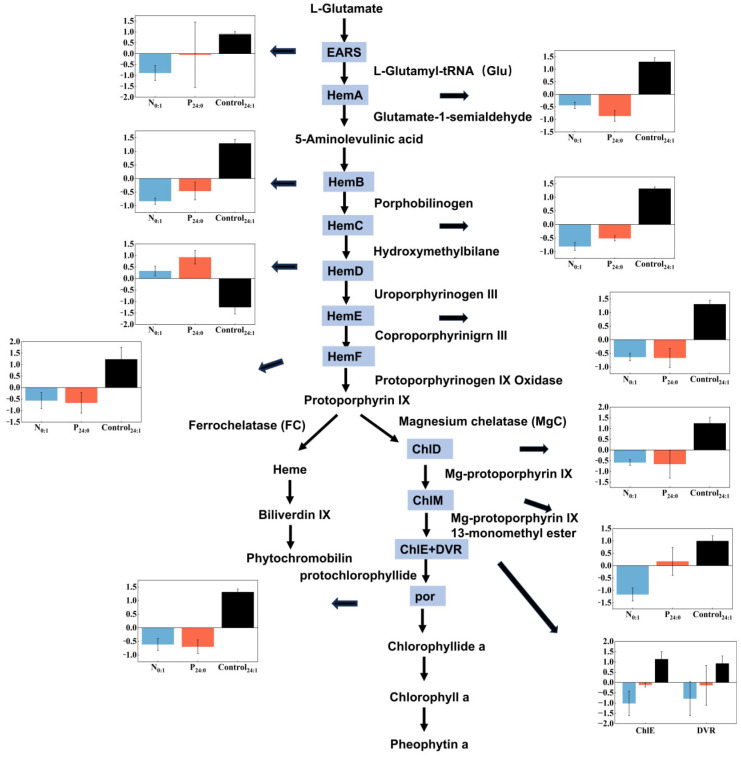
An annotated schematic of the porphyrin and chlorophyll biosynthetic pathway in the *Chattonella marina* complex N-limited (N_0:1_), P-limited (P_24:0_) and NP-sufficient (Control_24:1_) conditions. Data represent the mean ± SD, n = 3.

Pathway analysis (Figure 10) revealed a coordinated downregulation of genes across the porphyrin and chlorophyll biosynthesis pathways under both N and P limitation. Chlorophyll *a* biosynthesis proceeds through the tetrapyrrole pathway, which originates from L-glutamate via the C5 pathway [35,36,37]. Specifically, L-glutamate is first ligated to tRNA by glutamyl-tRNA synthetase (*EARS*) to form glutamyl-tRNA, which is subsequently reduced by glutamyl-tRNA reductase (*HemA*) to generate glutamate-1-semialdehyde (GSA). GSA is then converted into 5-aminolevulinic acid (ALA) by glutamate-1-semialdehyde aminotransferase (*HemL*). ALA, the universal precursor of tetrapyrroles, is condensed by ALA dehydratase (*HemB*) to form porphobilinogen (PBG), which is further polymerized by porphobilinogen deaminase (*HemC*) into hydroxymethylbilane (HMB). The linear tetrapyrrole HMB is subsequently cyclized by uroporphyrinogen III synthase (*HemD*) to yield uroporphyrinogen III (Uro III), a key branch-point intermediate in tetrapyrrole metabolism [38,39].

Uroporphyrinogen III is then decarboxylated by uroporphyrinogen decarboxylase (*HemE*) to produce coproporphyrinogen III, which undergoes oxidative decarboxylation via coproporphyrinogen oxidase (*HemF*) to form protoporphyrinogen IX. This intermediate is further oxidized by protoporphyrinogen oxidase into protoporphyrin IX (Proto IX). At this critical branching node, Proto IX is directed toward chlorophyll biosynthesis through the magnesium branch. In this branch, magnesium chelatase (*ChlD*) catalyzes the insertion of Mg^2+^ into Proto IX to generate Mg-protoporphyrin IX (Mg-Proto IX), which is subsequently methylated by Mg-protoporphyrin methyltransferase (*ChlM*) to form Mg-protoporphyrin IX monomethyl ester (MgPME). MgPME is then converted into protochlorophyllide (Pchlide) via the cyclase complex (including *ChlE*), followed by the light-dependent reduction of Pchlide to chlorophyllide *a* catalyzed by protochlorophyllide oxidoreductase (*por*). Finally, chlorophyllide *a* is esterified to produce chlorophyll *a*, the end product of the pathway [35,40].

It was evident that genes involved in the early steps of tetrapyrrole synthesis, from L-glutamate through *EARS*, to *HemC*, as well as those in the late steps from *HemE* to *HemF* were all downregulated. In contrast, the upregulation of *HemD*, which catalyzes the conversion of hydroxymethylbilane to uroporphyrinogen III, coupled with the downregulation of *HemE*, likely created a metabolic bottleneck, resulting in the accumulation of uroporphyrinogen III. Furthermore, the downstream branch leading to chlorophyll biosynthesis, from *ChlD* to *por*, also exhibited low expression levels, leading to reduced production of key intermediates, including Mg-protoporphyrin IX, Mg-protoporphyrin IX monomethyl ester, and protochlorophyllide, as well as the end product chlorophyll *a*. These patterns were consistent under both N and P limitation conditions.

## 3. Discussion

This study compared the effects of nitrogen (N) and phosphorus (P) limitation on the *Chattonella marina* complex. Both N and P limitation reduced growth, photosynthetic performance, and the expression of genes involved in tetrapyrrole and chlorophyll biosynthesis. A clear difference emerged in hemolytic activity and ROS levels: N limitation led to very low hemolytic activity and ROS levels, whereas P limitation left both relatively high, comparable to the nutrient-replete control. Adding the antioxidant NAC significantly lowered hemolytic activity, pointing to ROS as a contributing factor. Transcriptome data showed that under N limitation, genes for nitrogen uptake and initial reduction were upregulated, while downstream assimilation genes were downregulated. In contrast, under P limitation, all nitrogen-metabolism-related genes were downregulated. In the tetrapyrrole pathway, most genes were downregulated except *HemD* under both nutrient-depleted conditions, suggesting a possible bottleneck. Together, these results indicate that hemolytic toxicity is likely linked to ROS levels and disturbances in chlorophyll biosynthesis.

### 3.1. The Photosynthetic System of C. marina

Nitrogen (N) and phosphorus (P) are essential nutrients that regulate phytoplankton growth by controlling cellular stoichiometry (e.g., N:C ratio and chlorophyll content) and modulating key physiological processes such as photosynthesis and pigment biosynthesis [41]. Limitation of either nutrient has been widely reported to impair growth and photosynthetic performance in many microalgae [42,43,44,45,46], and similar effects were observed in *C. marina* (Figure 1, Figure 3 and Figure 4). However, the responses of growth and photosynthetic performance in *C. marina* differed between the two nutrient limitations. Nitrogen deprivation reduced the growth rate, whereas phosphorus limitation induced an earlier transition to the stationary phase, resulting in reduced biomass accumulation.

The decline in photosynthetic performance under N limitation can be attributed to its direct impact on protein synthesis [47,48] and chlorophyll production [49,50]. Nitrogen deficiency restricts the synthesis of key photosynthetic proteins and promotes the degradation of PSII components, such as the D1 protein and the CP47 chlorophyll–protein complex [44,47,51]. Consequently, the functionality of the PSII reaction center is compromised, leading to reduced photochemical efficiency, as reflected by decreases in F_v_/F_m_ and Y_II_ [48,52,53]. The responses of photosynthetic parameters under N limitation (Figure 3) in *C. marina* indicate a strong inhibition of PSII functionality. Both F_v_/F_m_ and Y_II_ decreased significantly (Figure 3A,B), particularly during the exponential phase, reflecting rapid impairment of photochemical efficiency. Similar responses have been widely reported in other microalgae, including diatoms and chlorophytes, where nitrogen limitation leads to a pronounced decline in PSII efficiency due to its direct involvement in protein synthesis and chlorophyll biosynthesis [48,51,54]. For example, in diatoms, nitrogen starvation rapidly suppresses photosynthetic protein turnover and electron transport, resulting in decreased F_v_/F_m_ and Y_II_ [48,54].

Phosphorus is a key component of ATP, nucleic acids, sugar phosphates, and membrane phospholipids; thus, its deficiency can restrict ATP-dependent metabolism, alter membrane structure, and disrupt the photosynthetic electron transport chain [55,56,57]. These changes may further constrain chlorophyll biosynthesis and carbon fixation, ultimately reducing photosynthetic efficiency [58]. Under P limitation, F_v_/F_m_ in *C. marina* also declined significantly, particularly in the stationary phase, which is consistent with observations in other microalgae, including diatoms and raphidophytes. For instance, reduced F_v_/F_m_ under phosphorus deficiency has been reported in *Thalassiosira weissflogii* and other marine phytoplankton, indicating impaired PSII efficiency under P stress [56,57,58,59,60]. However, compared to nitrogen limitation, the effect of phosphorus limitation on Y_II_ during the exponential phase in *C. marina* was less pronounced (Figure 3B), suggesting that phosphorus limitation exerts a more indirect and gradual effect on photosynthetic performance. This is likely because phosphorus primarily constrains cellular energy metabolism, including ATP production and membrane lipid remodeling, rather than directly limiting the synthesis of photosynthetic proteins.

### 3.2. Nutrient Stress and ROS in C. marina

Nutrient stress in *C. marina* markedly affects cellular physiology and photosynthetic performance (Figure 3), and previous studies have shown that photosynthetic activity in this species is closely linked to ROS production and redox regulation [27]. In this study, a greater proportion of absorbed light energy was dissipated via non-regulated pathways (Y_NO_), while regulated photoprotective dissipation (Y_NPQ_) remained comparatively low in *C. marina* (Figure 4). Under N limitation, Y_NPQ_ was significantly lower than in the other two treatment groups. This imbalance in energy allocation reflects a reduced capacity for controlled energy dissipation and indicates decreased susceptibility to oxidative stress under N-limited conditions (Figure 5). The consistently low ROS levels, together with reduced Y_NPQ_, support the interpretation of a suppressed photosynthetic metabolism, where low N availability limits electron transport chain components to such an extent that photoprotection is largely unnecessary. In diatoms, nitrogen starvation leads to a rapid decrease in photosynthetic efficiency, accompanied by a considerable increase in ROS levels and a reduction in photoprotective dissipation. These contrasts with *C. marina*, where ROS levels remain low (Figure 5) and Y_NPQ_ remains low (Figure 4A,D), likely due to a reduced reliance on photoprotective mechanisms. Additionally, green algae such as *Chlorella vulgaris* demonstrate a different response: nitrogen limitation causes a moderate decrease in photosynthetic efficiency but maintains a higher level of Y_NPQ_ for photoprotection [51]. This suggests that *C. marina* may have a different adaptive strategy, minimizing energy dissipation at the cost of decreased photosynthetic performance under nitrogen stress.

In contrast, under P limitation, *C. marina* produced high levels of ROS (Figure 5), accompanied by high Y_NPQ_ (Figure 4B,E) and a sustained growth rate (Figure 1), suggesting a state of energetic imbalance wherein ATP limitation drives electron chain over-reduction, requiring active photoprotection despite continued carbon assimilation. Compared to nitrogen limitation, which primarily affects protein synthesis and electron transport, phosphorus limitation appears to exert a more indirect effect, where energy balance becomes increasingly critical. Similar effects have been observed in other species, such as *Karlodinium veneficum*, where phosphorus deficiency induces a significant increase in ROS production along with enhanced Y_NPQ_, as the species compensates for reduced energy availability by increasing energy dissipation [61]. Furthermore, the synthesis of photoprotective pigments, including those involved in the xanthophyll cycle, is also inhibited under nutrient limitation, leading to reduced non-photochemical quenching (NPQ) capacity [62,63,64]. The decline in NPQ further exacerbates imbalances in energy dissipation, contributing to altered redox homeostasis.

ROS production in *C. marina* is primarily associated with two pathways: a cell surface-associated NADPH oxidase-like system and photosynthetic electron transport [53,65,66,67]. The NADPH oxidase system generates the superoxide anion (O_2_^−^·) through the reduction of molecular oxygen, which can subsequently be converted to hydrogen peroxide (H_2_O_2_) [65,68]. In parallel, photosynthetic electron transport may represent a major intracellular source of ROS, as the generation of superoxide appears to be closely linked to electron flow within the photosynthetic apparatus. This interpretation is supported by the finding that the inhibition of photosynthesis by DCMU leads to a significant reduction in ROS levels within a few hours of exposure [27,53,67]. Moreover, ROS production is typically higher under illuminated conditions and during active growth phases, for example, under P-limited conditions (Figure 5), further supporting the dependence of ROS generation on photosynthetic activity [69].

At the molecular level, nutrient limitation is known to induce coordinated downregulation of genes associated with photosynthetic complexes, electron transport chains, and pigment biosynthesis [70]. Under nitrogen limitation, reduced synthesis of chlorophyll and key PSII proteins, particularly the D1 reaction center protein, impairs PSII repair and accelerates photoinhibition [71,72]. In the present study, no evidence of enhanced ROS production was observed under nitrogen limitation. Instead, ROS levels remained consistently low, accompanied by reduced Y_NPQ_. This pattern suggests that nitrogen limitation severely constrained photosynthetic electron transport. This disruption of PSII turnover weakens electron transport capacity and consequently limits ROS generation via photosynthetic pathways.

Phosphorus limitation affects photosynthesis through distinct mechanisms, primarily by constraining ATP-dependent metabolism and altering membrane phospholipid composition, both of which are essential for maintaining efficient photosynthetic electron transport [55,56,57] In the present study, phosphorus limitation in *C. marina* led to a significant decline in photosynthetic performance, as evidenced by reduced F_v_/F_m_ and Y_II_ values (Figure 3A,B). Despite the suppression of photochemical efficiency, the production of ROS increased significantly (Figure 5), and Y_NPQ_ values rose accordingly (Figure 4B,E). This pattern may indicate that phosphorus limitation triggers photoinhibition and excessive excitation stress, rather than merely a downregulation of photosynthetic activity.

Collectively, these results indicate that both N and P limitation alter photosynthetic energy allocation, suppress electron transport, and impair photoprotective processes, thus modulating ROS production in *C. marina*.

### 3.3. Hemolytic Activity and ROS in C. marina

Hemolytic activity and ROS production in *C. marina* were markedly induced under P-limited conditions (Figure 2 and Figure 5). Together with the evidence that addition of NAC significantly reduced hemolytic activity (Figure 6), these results indicate that the toxicity of *C. marina* is driven synergistically by hemolytic toxins and ROS.

Given that porphyrin-related compounds have been proposed as potential structural analogs or precursors of hemolytic toxins in *C. marina* [18,19,20,21,22,23,24,25,26,27,28,29,30,31,32,33,34,35,36,37,38,39,40,41,42,43,44,45,46,47,48,49,50,51,52,53,54,55,56,57,58,59,60,61,62,63,64,65,66,67,68,69,70,71,72,73], the porphyrin and chlorophyll biosynthesis pathways were further investigated. Transcriptomic analysis revealed that, compared with the control, both N and P limitation suppressed the overall activity of the tetrapyrrole biosynthesis pathway. Specifically, the production of 5-aminolevulinic acid (ALA), the universal precursor of tetrapyrroles, was reduced, leading to decreased formation of downstream intermediates such as porphobilinogen and hydroxymethylbilane. However, some bioactive compounds, i.e., Uro III or other porphyrin intermediates in tetrapyrrole metabolism, exhibited an opposite trend. This inconsistency between sequential enzymatic steps suggests a disruption in pathway coordination, resulting in metabolic imbalance. Due to the downregulation of downstream enzymes, particularly those involved in the conversion of Uro III, the transformation into coproporphyrinogen III and protoporphyrinogen IX may be constrained, potentially leading to the accumulation of intermediate porphyrin compounds. Meanwhile, key intermediates in the downstream branch toward chlorophyll biosynthesis, including Mg-protoporphyrin IX, Mg-protoporphyrin IX monomethyl ester, and protochlorophyllide, were all reduced, indicating an overall suppression of chlorophyll synthesis and a consequent decrease in chlorophyll *a* production.

These intermediate porphyrin compounds, including uroporphyrin, coproporphyrin, and protoporphyrin derivatives, possess well-established photodynamic properties. Under illumination, they can generate ROS and induce oxidative damage to biological membranes [74,75]. Therefore, they are considered to have potential cytotoxic effects and may contribute to hemolytic activity.

Surprisingly, those potential hemolytic compounds were induced under both N and P limitation, yet high hemolytic activity was observed only under P limitation (Figure 2). Thus, it is likely that those compounds alone were insufficient to cause hemolysis; their action requires the presence of ROS. Conversely, ROS themselves are not solely responsible for hemolytic activity [14,27,76,77]. Therefore, the hemolytic activity observed in this study likely results from a combined effect of the porphyrin derivatives and ROS. This conclusion is further supported by the NAC experiments, in which the antioxidant (NAC) significantly reduced hemolytic activity of *C. marina* (Figure 6). Similar evidence for the synergistic role of ROS has been reported previously. In *C. marina*, ichthyotoxicity has been explicitly attributed to the synergistic action of ROS and free fatty acids, rather than to either factor alone [14].

Due to its role as a redox buffer and its low toxicity, NAC can be added to culture media to moderately promote the growth rate of the diatoms *Chaetoceros calcitrans* and *C. muelleri*, but it has no effect on *Skeletonema costatum* [78]. NAC further induced the production of eicosapentaenoic acid (EPA), a potential bioactive compound [14], in the diatom *Thalassiosira pseudonana* and haptophytes *Pavlova salina* [78]. Marshall [14] reported that the mechanism of ichthyotoxicity in *C. marina* may involve ROS-mediated oxidation of high amounts of EPA. Similarly, non-PST (paralytic shellfish toxins)-producing *Alexandrium tamarense* strain had a reduced toxic effect on ciliate *Tiarina fusus* and dinoflagellate *Polykrikos kofoidii* upon addition of antioxidant compounds, including peroxidase, superoxide dismutase (SOD), or trypsin [79], indicating that the toxicity of non-PST-producing *Alexandrium* to protists may result from ROS-mediated oxidation of polyunsaturated fatty acids (e.g., EPA) or other secondary compounds. Meanwhile, Flores [79] also found that the addition of catalase did not increase the survival of *P. kofoidii* when exposed to either PST-producing or non-PST-producing *Alexandrium*, indicating the types or dosages of antioxidant compounds function differently in regulating the ROS production, thereby altering the synergistically effect of ROS and/or bioactive compounds.

Notably, the persistence of hemolytic activity under phosphorus limitation, combined with its near absence under nitrogen limitation, indirectly supports the hypothesis that the hemolytic toxins of *C. marina* are nitrogen-containing compounds whose biosynthesis depends on nitrogen availability [80,81].

## 4. Conclusions

This study examined the physiological and toxicological responses of the *Chattonella marina* complex to N and P limitation. Both limitations reduced growth, photosynthesis, and expression of tetrapyrrole/chlorophyll pathway genes. However, N limitation strongly suppressed photosynthetic electron transport, ROS, and hemolytic activity, whereas P limitation maintained high ROS and hemolytic activity despite reduced growth. A metabolic bottleneck in the tetrapyrrole pathway (most genes downregulated except *HemD*) likely leads to accumulation of photoactive porphyrin intermediates. The antioxidant NAC lowered hemolytic activity, whereas high toxicity occurred under P limitation and nutrient-sufficient conditions where ROS levels were elevated, indicating a synergistic action between ROS and porphyrin-like compounds. Under N limitation, low ROS and constrained synthesis of nitrogen-containing toxins explain the very low hemolytic activity. Thus, hemolytic toxicity depends on both nitrogen availability and cellular redox status. These findings show that P-limited, N-replete conditions favor high toxicity, posing greater ecological risk. Future metabolomic and biochemical studies are needed to identify the specific compounds and their interactions with ROS.

## 5. Materials and Methods

### 5.1. Culture Conditions

The strain of *Chattonella* spp. used in this study was originally isolated from the East China Sea near Zhoushan, Zhejiang Province, in the summer of 2017 and had been maintained in a laboratory since then. The strain was identified morphologically as *C. antiqua* [82] and molecularly as belonging to the *Chattonella marina* complex [1]; hereafter, it is referred to as *C. marina* for brevity. Cultures were maintained at 25 °C, under a light intensity of 50 μmol photons m^−2^ s^−1^ (12 h:12 h light–dark cycle), in f/2 medium [83] at a salinity of 30. For each generation, a 50 mL aliquot of the algal culture in the exponential growth phase (approximately days 7–9), with a density of about 1 × 10^4^ cells mL^−1^, was inoculated into 200 mL of fresh f/2 medium, resulting in an initial inoculation density of approximately 1 × 10^3^ cells mL^−1^.

### 5.2. Experimental Setup—Different NP Ratios

The experiment employed a series of modified f/2 media, with varying NaNO_3_-N and NaH_2_PO_4_-P concentrations, while all other components remained consistent with the standard f/2 formulation (Table 1).

A 50 mL aliquot of exponentially growing *C. marina* (~5 × 10^3^ cells mL^−1^) from the stock culture was inoculated into 200 mL of fresh medium prepared as described above, resulting in an initial cell density of approximately 1.0 × 10^3^ cells mL^−1^. All other experimental conditions were the same as the maintenance conditions, and each treatment was set up in triplicate.

Throughout the culture period, subsamples for cell counting (5 mL) and hemolytic activity (5 mL) analysis were collected daily in all treatments. Photosynthetic parameters (3 mL) and reactive oxygen species (ROS, 5 mL) were measured daily only for treatment of N-limited (N_0:1_), P-limited (P_24:0_) and control (Control_24:1_) conditions. Subsamples for transcriptomic analysis (50 mL) were conducted at the end of the exponential phase (day 5) of *C. marina* in treatment of N_0:1_, P_24:0_ and Control_24:1_.

### 5.3. Data Analysis

#### 5.3.1. Photosynthetic Growth

Algal samples were collected two hours after the start of the daily light period. A 1 mL aliquot of the culture was then fixed with 20 μL of Lugol’s iodine solution [84]. Cells were enumerated under an optical microscope (Chongqing Optec Instrument Co., Ltd., Chongqing, China) at 40× magnification using a 1 mL sample placed in a 0.1 mL Sedgwick Rafter counting chamber. The average growth rate (μ) was calculated using the following formula [85]:


$$\mu =\frac{In(N2/N1)}{t2-t1}$$

N1 and N2 are the algal cell density during the early and late logarithmic growth phase, and t1 and t2 are the corresponding time points.

#### 5.3.2. Photosynthetic Activity

Photosynthetic activity was assessed using a chlorophyll fluorescence system (MAXI-IMAGING PAM, Walz, Effeltrich, Germany). Three mL of subsamples were transferred to a black 24-well plate. Seawater was set as a blank control. Following a 20 min dark adaptation period, fluorescence induction kinetics and rapid light-response curves were measured using measuring light of 1.5–2.5 μmol m^−2^ s^−1^ and actinic light of 395 μmol m^−2^ s^−1^. The parameters obtained included the maximum quantum yield of photosystem II (F_v_/F_m_, F_v_/F_m_ = (F_m_ − F_0_)/F_m_), the effective quantum yield (Y_II_ = (F_m_′ − F_t_)/F_m_′), the quantum yields of regulated (Y_NPQ_ = F_t_/F_m_’ − F_t_/F_m_) and non-regulated (Y_NO_ = F_t_/F_m_) energy dissipation (Y_II_ + Y_NPQ_ + Y_NO_ = 1). F_0_, F_m_, F_m_’, F_t_ and F_v_ (calculated as F_m_ − F_0_) represented as the minimum fluorescence, maximum fluorescence, actual fluorescence, instantaneous fluorescence, and the variable fluorescence, respectively. The relative electron transport rate (rETR) was measured under light intensities ranging from 0 to 1252 μmol m^−2^ s^−1^, and the maximum relative electron transport rate (rETR_max_) was calculated using the rapid light-response curves.

#### 5.3.3. Hemolytic Activity

Hemolytic activity was determined using a modified rabbit blood assay [86,87]. A volume of 5 mL of the culture of *C. marina* was harvested by centrifugation at 8000× *g* for 10 min at 4 °C. The pellet was resuspended in ELA buffer (8.775 g/L NaCl, 0.238 g/L KCl, 0.307 g/L MgSO_4_·7H_2_O, 0.551 g/L CaCl_2_·2H_2_O, and 1.476 g/L TRIS, adjusted with 0.1 M HCl to pH of 7.4) and disrupted by sonication (JY92-IIN homogenizer, 30% power, 3 min with cycles of 2 s on and 1 s off) to obtain the extract. All steps were performed on ice or at 4 °C. The mixture of 1 mL of toxin extract and 1 mL of the prewashed erythrocyte (Shanghai Guduo Biological Technology Co., Ltd., Shanghai, China, 5 × 10^7^ cells mL^−1^) was used as a test sample (E_414_). Mixtures of 1 mL of ELA buffer and 1 mL of prewashed blood cells served as the blank control (A_414_), while toxin extract (1 mL) and ELA buffer (1 mL) served as the negative control (N_414_). Erythrocytes (1 mL) lysed with 1% Triton X-100 (900 μL of ELA buffer, and 100 μL of Triton X-100, Shanghai Yuan Mu Biotechnology Co., Ltd., Shanghai, China) served as the positive control (P_414_). All samples were incubated 5 h under the same algal culture conditions, and centrifuged at 2000× *g* for 5 min at 25 °C. Each supernatant (200 μL) was transferred to a 96-well microplate, and the released homoglobin absorbance was measured at 414 nm in a microplate reader (Infinite M1000 Pro, Männedorf, Switzerland). Then, the hemolytic activity was calculated according to Ling and Trick [86].

$$\;\mathrm{Hemolytic}\;\mathrm{activity}\;\left(\%\right)=\frac{E414-A414-N414}{P414}\times 100\%$$

where E_414_, A_414_, N_414_ and P_414_ are the absorbances at 414 nm of the test samples, blank control, negative control and positive control, respectively.

The half-effective concentration (EC_50_) of *C. marina* was determined by a concentration–response curve using cells grown in the exponential stage (Figure S1). A final concentration of 500 cells mL^−1^ was used thereafter in the present study to quantify the hemolytic activity.

#### 5.3.4. Reactive Oxygen Species Assay

Reactive oxygen species (ROS) levels in *C. marina* were quantified following the method of the cell-permeable fluorogenic probe 2′,7′-dichlorodihydrofluorescein diacetate (H_2_DCFDA) [88]. A 10 mM H_2_DCFDA stock solution in anhydrous DMSO was prepared, and adjusted to 10 μM in each culture medium. Subsamples of *C. marina* in each treatment (with a uniform final density of 500 cells mL^−1^) were placed in a 96-well plate. To each well, 10 μM H_2_DCFDA working solution was added to a final volume of 200 µL. A solvent control containing only H_2_DCFDA was included. The plate was incubated for 30 min under a light intensity of 15 μmol m^−2^ s^−1^. Fluorescence was then measured using a microplate reader (Infinite M1000 Pro, Männedorf, Switzerland) with excitation and emission wavelengths of 488 nm and 525 nm, respectively. Results are expressed as absolute fluorescence units (FU).

#### 5.3.5. Synergistical Effect of ROS

To evaluate the combined effects of ROS and potential hemolytic toxins, N-Acetyl-L-cysteine (NAC, Sangon Biotech, Shanghai, China) was used as an anti-oxidative compound to modulate their synergistic interaction. A stock solution of NAC at a concentration of 500 mM was prepared by dissolving it in sterile deionized water. The solution was sterilized by filtration through a 0.22 μm Nylon membrane (Tianjin Jinteng Experiment Equipment Co., Ltd., Tianjing, China) and added to rabbit erythrocytes to achieve final concentrations of 0, 0.05, and 0.1 mM. The treated erythrocytes were subsequently mixed with algal lysates (obtained by ultrasonication in an ice bath) and incubated for 5 h under a light intensity of 50 μmol photons m^−2^ s^−1^.

#### 5.3.6. Transcriptomic Analysis

For transcriptomic analysis, 50 mL of *C. marina* from each treatment was collected during the logarithmic growth phase and centrifuged at 8000× *g* for 10 min. The pellet was immediately frozen and shipped on dry ice to Lianchuan Biotech (LC-Bio Technologies) Co., Ltd., Hangzhou, China) for RNA sequencing.

Total RNA was extracted using TRIzol reagent (Thermo Fisher Scientific, Waltham, MA, USA, 15596018) according to the manufacturer’s instructions. RNA concentration and purity were assessed using a Qubit 3.0 Fluorometer (Thermo Fisher Scientific, Waltham, MA, USA, Q33216), while RNA integrity was evaluated with an Agilent 5300 Fragment Analyzer (Agilent Technologies, Santa Clara, CA, USA, M5311AA). Only high-quality RNA samples with an RNA integrity number (RIN) greater than 7.0 were used for subsequent library construction. For mRNA enrichment, 2 μg of total RNA was subjected to two rounds of purification using mRNA Capture Beads 2.0 (Yeasen Biotechnology Co., Ltd., Cat. 12629ES, Shanghai, China). The purified mRNA was then fragmented into short fragments in the presence of magnesium ions at 94 °C (Yeasen Biotechnology, Cat. 12340ES97, Shanghai, China). These RNA fragments were reverse-transcribed to generate first-strand cDNA using reverse transcriptase, followed by second-strand cDNA synthesis using *E. coli* DNA polymerase I, RNase H, and dUTP solution (Yeasen Biotechnology, Cat. 12340ES97, Shanghai, China). Subsequently, an adenine base was added to the 3′ ends of the double-stranded cDNA fragments to facilitate adapter ligation. Indexed adapters with thymine overhangs were ligated to the A-tailed fragments. Dual-index adapters were used, and the ligation products were amplified by PCR under the following conditions: initial denaturation at 98 °C for 1 min; 14 cycles of 98 °C for 10 s, 60 °C for 30 s, and 72 °C for 30 s; followed by a final extension at 72 °C for 5 min. The resulting cDNA libraries had an average insert size of 400 ± 50 bp. Strand specificity was achieved during PCR amplification by selectively amplifying cDNA strands lacking uracil using a high-fidelity DNA polymerase. PCR products were purified using Hieff NGS DNA Selection Beads (Yeasen Biotechnology, Cat. 12601ES75, Shanghai, China). Finally, paired-end sequencing (2 × 150 bp) was performed on an Illumina NovaSeq^TM^ X Plus platform (LC-Bio Technology Co., Ltd., Hangzhou, China) following the manufacturer’s protocols.

Raw reads were processed using Cutadapt (v1.9) to remove adapters, low-quality reads, and reads containing poly-A/poly-G or ambiguous bases. Clean reads were assessed using FastQC (v0.11.9) and aligned to the reference genome using HISAT2 (v2.2.1). Transcript assembly and quantification were performed using StringTie (v2.1.6) and Ballgown (v3.23), and gene expression levels were calculated as FPKM values. Differential expression analysis was conducted using DESeq2 (v1.48.1) or edgeR (v3.40.2), with genes meeting the criteria of FDR < 0.05 and |fold change| ≥ 2 defined as differentially expressed genes (DEGs). Functional enrichment analyses, including Gene Ontology (GO) and KEGG pathway analyses, were performed with *p* < 0.05 as the significance threshold. In addition, gene set enrichment analysis (GSEA) was conducted using GSEA software (v4.1.0). Alternative splicing events were identified using rMATS (v4.1.1), and SNPs were detected using SAMtools (v1.9) and annotated with ANNOVAR (release 2013-08-23).

### 5.4. Statistical Analysis

Data analysis and visualization were conducted using the software Origin 26 and SPSS 27.0. The effects of different N:P ratios on the growth, photosynthetic activity, PSII energy distribution, hemolytic activity, and ROS of *C. marina* were assessed using one-way analysis of variance (ANOVA), with statistical significance set at *p* < 0.05. Transcriptomic data, specifically gene expression levels, were visualized as heatmaps using the OmicStudio tools at v3.6 https://www.omicstudio.cn/tool (accessed on 15 December 2025).

## Figures and Tables

**Table 1:** Nutrient status (N and P) of *C. marina* during the experiment.

ID	N (ca. μmol·L^−1^)	P (ca. μmol·L^−1^)	NP Ratio
Control_24:1_	882	36.2	24:1
NP_12:1_	441	36.2	12:1
NP_6:1_	221	36.2	6:1
NP_2.4:1_	88	36.2	2.4:1
NP_1.2:1_	44	36.2	1.2:1
N_0:1_	0.0	36.2	0:1
P_24:0_	882	0.0	24:0
NP_480:1_	882	1.8	480:1
NP_240:1_	882	3.6	240:1
NP_96:1_	882	9.1	96:1
NP_48:1_	882	18.1	48:1

## Data Availability

The original contributions presented in this study are included in the article/Supplementary Materials. Further inquiries can be directed to the corresponding author.

## Supplementary Materials

The following supporting information can be downloaded at: https://www.mdpi.com/article/10.3390/toxins18050226/s1, Figure S1: The half-effective concentration (EC_50_) of *C. marina;* Figure S2: Hemolytic activity of *C. marina* under different stoichiometry conditions; Figure S3: KEGG enrichment analysis of metabolic pathways for differentially expressed genes in *C. marina* under N-limited (N_0:1_) and NP-sufficient (Control_24:1_) conditions; Figure S4: KEGG enrichment analysis of metabolic pathways for differentially expressed genes in *C. marina* under P-limited (P_24:0_) and NP-sufficient (Control_24:1_) conditions; Figure S5: KEGG enrichment analysis of metabolic pathways for differentially expressed genes in *C. marina* under N-limited (N_0:1_), P-limited (P_24:0_) and NP-sufficient (Control_24:1_) conditions.

## References

[1] Min J., Kim K.Y. (2022). Quantification of the ichthyotoxic raphidophyte *Chattonella marina* complex by applying a droplet digital PCR. Algae.

[2] Imai I., Yamaguchi M. (2012). Life cycle, physiology, ecology and red tide occurrences of the fish-killing raphidophyte *Chattonella*. Harmful Algae.

[3] Hiroishi S., Okada H., Imai I., Yoshida T. (2005). High toxicity of the novel bloom-forming species *Chattonella ovata* (Raphidophyceae) to cultured fish. Harmful Algae.

[4] Cortés-Altamirano R., Alonso R., Sierra A. (2006). Fish mortality associated with *Chattonella marina* and *C.* cf. *ovata* (Raphidophyceae) blooms in Sinaloa (Mexico). Harmful Algae News.

[5] García-Mendoza E., Cáceres-Martínez J., Rivas D., Fimbres-Martinez M., Sánchez-Bravo Y., Vásquez-Yeomans R., Medina-Elizalde J. (2018). Mass mortality of cultivated northern bluefin tuna *Thunnus thynnus orientalis* associated with *Chattonella* species in Baja California, Mexico. Front. Mar. Sci..

[6] Liao Z., Wang Z. (2019). Interspecies competition between *Chattonella marina* and three typical marine diatoms. Mar. Environ. Sci..

[7] Sakamoto S., Lim W.A., Lu D., Dai X., Orlova T., Iwataki M. (2021). Harmful algal blooms and associated fisheries damage in East Asia: Current status and trends in China, Japan, Korea and Russia. Harmful Algae.

[8] Padmakumar K.B., Thomas L.C., Salini T.C., John E.M., Menon N.R., Sanjeevan V.N. (2011). Monospecific bloom of noxious raphidophyte *Chattonella marina* in the coastal water of south west coast of India. Int. J. Biosci..

[9] Hallegraeff G.M., Schweibold L., Jaffrezic E., Rhodes L., MacKenzie L., Hay B., Farrell H. (2021). Overview of Australian and New Zealand harmful algal species occurrences and their societal impacts in the period 1985 to 2018, including a compilation of historic records. Harmful Algae.

[10] Marshall J.A., Hallegraeff G. (1999). Comparative ecophysiology of the harmful alga *Chattonella marina* (Raphidophyceae) from South Australian and Japanese waters. J. Plankton Res..

[11] Zhang Y., Fu F.-X., Whereat E., Coyne K.J., Hutchins D.A. (2006). Bottom-up controls on a mixed-species HAB assemblage: A comparison of sympatric *Chattonella subsalsa* and *Heterosigma akashiwo* (Raphidophyceae) isolates from the Delaware Inland Bays, USA. Harmful Algae.

[12] Anderson D.M., Fensin E., Gobler C.J., Hoeglund A.E., Hubbard K.A., Kulis D.M., Landsberg J.H., Lefebvre K.A., Provoost P., Richlen M.L. (2021). Marine harmful algal blooms (HABs) in the United States: History, current status and future trends. Harmful Algae.

[13] Qi Y., Huang C. (1997). The causative mechanism of *Chattonella marina* bloom in Dapeng Bay, the south China Sea. Oceanol. Limnol. Sin..

[14] Marshall J.-A., Nichols P.D., Hamilton B., Lewis R.J., Hallegraeff G.M. (2003). Ichthyotoxicity of *Chattonella marina* (Raphidophyceae) to damselfish (*Acanthochromis polycanthus*): The synergistic role of reactive oxygen species and free fatty acids. Harmful Algae.

[15] Gao L., Yang W., Liu J., Li H. (2009). Progress in the study of toxicological mechanism of *Chattonella marina*. Mar. Sci. Bull..

[16] Endo M., Onoue Y., Kuroki A. (1992). Neurotoxin-induced cardiac disorder and its role in the death of fish exposed to *Chattonella marina*. Mar. Biol..

[17] Khan S., Ahmed M.S., Arakawa O., Onoue Y. (1995). Properties of neurotoxins separated from a harmful red tide organism *Chattonella marina*. Isr. J. Aquac..

[18] Kuroda A., Nakashima T., Yamaguchi K., Oda T. (2005). Isolation and characterization of light-dependent hemolytic cytotoxin from harmful red tide phytoplankton *Chattonella marina*. Comp. Biochem. Physiol. C.

[19] Shimada M., Murakami T.H., Imahayashi T., Ozaki H.S., Toyoshima T., Okaichi T. (1983). Effects of sea bloom, *Chattonella antiqua*, on gill primary lamellae of the young yellowtail, *Seriola quinqueradiata*. Acta Histochem. Cytochem..

[20] Endo M., Sakai T., Kuroki A. (1985). Histological and histochemical changes in the gills of the yellowtail *Seriola quinqueradiata* exposed to the Raphidophycean flagellate *Chattonella marina*. Mar. Biol..

[21] Sakai T., Yamamoto K.i., Endo M., Kuroki A., Kumanda K., Takeda K., Aramaki T. (1986). Changes in the gill carbonic anhydrase activity of fish exposed to *Chattonella marina* red tide, with special reference to the mortality. Nippon Suisan Gakkaishi.

[22] Tanaka K., Muto Y., Shimada M. (1994). Generation of superoxide anion radicals by the marine phytoplankton organism, *Chattonella antiqua*. J. Plankton Res..

[23] Ishimatsu A., Oda T., Yoshida M., Ozaki M. (1996). Oxygen radicals are probably involved in the mortality of yellowtail by *Chattonella marina*. Fish. Sci..

[24] Marshall J.-A., Ross T., Pyecroft S., Hallegraeff G. (2005). Superoxide production by marine microalgae. Mar. Biol..

[25] Marshall J.A., de Salas M., Oda T., Hallegraeff G. (2005). Superoxide production by marine microalgae: I. Survey of 37 species from 6 classes. Mar. Biol..

[26] Cho K., Ueno M., Liang Y., Kim D., Oda T. (2022). Generation of Reactive Oxygen Species (ROS) by Harmful Algal Bloom (HAB)-Forming Phytoplankton and Their Potential Impact on Surrounding Living Organisms. Antioxidants.

[27] Wu N., Tong M., Gou S., Zeng W., Xu Z., Jiang T. (2021). Hemolytic Activity in Relation to the Photosynthetic System in *Chattonella marina* and *Chattonella ovata*. Mar. Drugs.

[28] Cao J., Huan Q., Wu N., Jiang T. (2015). Effects of temperature, light intensity and nutrient condition on the growth and hemolytic activity of six species of typical ichthyotoxic algae. Mar. Environ. Sci..

[29] Huang J., Yang W., Liu J., Li H., Liu B. (2009). Effects of temperature, salinity, and light intensity on the grow and toxin production of *Chattonella marina*. Chin. J. Appl. Ecol..

[30] Conley D.J., Paerl H.W., Howarth R.W., Boesch D.F., Seitzinger S.P., Havens K.E., Lancelot C., Likens G.E. (2009). Controlling eutrophication: Nitrogen and phosphorus. Science.

[31] Lazzari P., Solidoro C., Salon S., Bolzon G. (2016). Spatial variability of phosphate and nitrate in the Mediterranean Sea: A modeling approach. Deep Sea Res. I.

[32] Yuan Z., Browning T.J., Zhang R., Wang C., Du C., Wang Y., Chen Y., Liu Z., Liu X., Shi D. (2023). Potential drivers and consequences of regional phosphate depletion in the western subtropical North Pacific. Limnol. Oceanogr. Lett..

[33] Yuan Z., Achterberg E.P., Engel A., Dai M., Browning T.J. (2024). Switches between nitrogen limitation and nitrogen–phosphorus co-limitation in the subtropical North Atlantic Ocean. Limnol. Oceanogr..

[34] Li T., Liu S., Huang L., Xu Z. (2005). Studies on phytoplankton community change at daya bay during a red tide. J. Trop. Oceanogr..

[35] Tanaka R., Tanaka A. (2007). Tetrapyrrole Biosynthesis in Higher Plants. Annu. Rev. Plant Biol..

[36] Fujita Y., Tsujimoto R., Aoki R. (2015). Evolutionary Aspects and Regulation of Tetrapyrrole Biosynthesis in Cyanobacteria under Aerobic and Anaerobic Environments. Life.

[37] Dailey H.A., Dailey T.A., Gerdes S., Jahn D., Jahn M., O’Brian M.R., Warren M.J. (2017). Prokaryotic Heme Biosynthesis: Multiple Pathways to a Common Essential Product. Microbiol. Mol. Biol. Rev..

[38] Yin L., Bauer C.E. (2013). Controlling the delicate balance of tetrapyrrole biosynthesis. Philos. Trans. R. Soc. B Biol. Sci..

[39] Wittmann D., Sinha N., Grimm B. (2021). Thioredoxin-dependent control balances the metabolic activities of tetrapyrrole biosynthesis. Biol. Chem..

[40] Wang P., Ji S., Grimm B. (2022). Post-translational regulation of metabolic checkpoints in plant tetrapyrrole biosynthesis. J. Exp. Bot..

[41] Geider R., Macintyre H.L., Graziano L., McKay R.M. (1998). Responses of the photosynthetic apparatus of *Dunaliella tertiolecta* (Chlorophyceae) to nitrogen and phosphorus limitation. Eur. J. Phycol..

[42] Brussaard C.P.D., Noordeloos A.A.M., Riegman R. (1997). Autolysis kinetics of the marine diatom *Ditylum brightwellii* (Bacillariophyceae) under nitrogen and phosphorus limitation and starvation. J. Phycol..

[43] Weers P.M.M., Gulati R.D. (1997). Growth and reproduction of *Daphnia galeata* in response to changes in fatty acids, phosphorus, and nitrogen in *Chlamydomonas reinhardtii*. Limnol. Oceanogr..

[44] Berges J.A., Falkowski P.G. (1998). Physiological stress and cell death in marine phytoplankton: Induction of proteases in response to nitrogen or light limitation. Limnol. Oceanogr..

[45] John E.H., Flynn K.J. (2000). Growth dynamics and toxicity of *Alexandrium fundyense* (Dinophyceae): The effect of changing N:P supply ratios on internal toxin and nutrient levels. Eur. J. Phycol..

[46] Bidle K.D., Falkowski P.G. (2004). Cell death in planktonic, photosynthetic microorganisms. Nat. Rev. Microbiol..

[47] Kolber Z., Zehr J., Falkowski P. (1988). Effects of Growth Irradiance and Nitrogen Limitation on Photosynthetic Energy Conversion in Photosystem II 1. Plant Physiol..

[48] Liefer J.D., Garg A., Campbell D.A., Irwin A.J., Finkel Z.V. (2018). Nitrogen starvation induces distinct photosynthetic responses and recovery dynamics in diatoms and prasinophytes. PLoS ONE.

[49] Ikaran Z., Suárez-Alvarez S., Urreta I., Castañón S. (2015). The effect of nitrogen limitation on the physiology and metabolism of *Chlorella vulgaris* var L3. Algal Res..

[50] Li Z., Lan T., Zhang J., Gao K., Beardall J., Wu Y. (2021). Nitrogen Limitation Decreases the Repair Capacity and Enhances Photoinhibition of Photosystem II in a Diatom. Photochem. Photobiol..

[51] Hockin N.L., Mock T., Mulholland F., Kopriva S., Malin G. (2012). The response of diatom central carbon metabolism to nitrogen starvation is different from that of green algae and higher plants. Plant Physiol..

[52] Long M., Tallec K., Soudant P., Le Grand F., Donval A., Lambert C., Sarthou G., Jolley D.F., Hégaret H. (2018). Allelochemicals from *Alexandrium minutum* induce rapid inhibition of metabolism and modify the membranes from *Chaetoceros muelleri*. Algal Res..

[53] Yuasa K., Shikata T., Kitatsuji S., Yamasaki Y., Nishiyama Y. (2020). Extracellular secretion of superoxide is regulated by photosynthetic electron transport in the noxious red-tide-forming raphidophyte *Chattonella antiqua*. J. Photochem. Photobiol. B.

[54] Negi S., Barry A.N., Friedland N., Sudasinghe N., Subramanian S., Pieris S., Holguin F.O., Dungan B., Schaub T., Sayre R. (2016). Impact of nitrogen limitation on biomass, photosynthesis, and lipid accumulation in *Chlorella sorokiniana*. J. Appl. Phycol..

[55] Carstensen A., Herdean A., Schmidt S.B., Sharma A., Spetea C., Pribil M., Husted S. (2018). The impacts of phosphorus deficiency on the photosynthetic electron transport chain. Plant Physiol..

[56] Lovio-Fragoso J.P., de Jesús-Campos D., López-Elías J.A., Medina-Juárez L.Á., Fimbres-Olivarría D., Hayano-Kanashiro C. (2021). Biochemical and Molecular Aspects of Phosphorus Limitation in Diatoms and Their Relationship with Biomolecule Accumulation. Biology.

[57] Bossa R., Di Colandrea M., Salbitani G., Carfagna S. (2024). Phosphorous Utilization in Microalgae: Physiological Aspects and Applied Implications. Plants.

[58] Kumari K., Samantaray S., Sahoo D., Tripathy B.C. (2021). Nitrogen, phosphorus and high CO2 modulate photosynthesis, biomass and lipid production in the green alga *Chlorella vulgaris*. Photosynth. Res..

[59] Liu S., Guo Z., Li T., Huang H., Lin S. (2011). Photosynthetic efficiency, cell volume, and elemental stoichiometric ratios in *Thalassirosira weissflogii* under phosphorus limitation. Chin. J. Oceanol. Limnol..

[60] Qi H., Wang J., Wang Z. (2013). A comparative study of maximal quantum yield of photosystem II to determine nitrogen and phosphorus limitation on two marine algae. J. Sea Res..

[61] Cui Y., Zhang H., Lin S. (2017). Enhancement of non-photochemical quenching as an adaptive strategy under phosphorus deprivation in the dinoflagellate *Karlodinium veneficum*. Front. Microbiol..

[62] Müller P., Li X.-P., Niyogi K.K. (2001). Non-photochemical quenching.A response to excess light energy. Plant Physiol..

[63] Montuori E., Lima S., Marchese A., Scargiali F., Lauritano C. (2024). Lutein Production and Extraction from Microalgae: Recent Insights and Bioactive Potential. Int. J. Mol. Sci..

[64] Yang Y., Li W., Li Y., Xu N. (2024). Effects of ocean acidification and nitrogen limitation on the growth and photophysiological performances of marine macroalgae *Gracilariopsis lemaneiformis*. Front. Mar. Sci..

[65] Kim D., Nakamura A., Okamoto T., Komatsu N., Oda T., Iida T., Ishimatsu A., Muramatsu T. (2000). Mechanism of superoxide anion generation in the toxic red tide phytoplankton *Chattonella marina*: Possible involvement of NAD(P)H oxidase. Biochim. Biophys. Acta.

[66] Yuasa K., Ichikawa T., Ishikawa Y., Jimbo H., Kawai-Yamada M., Shikata T., Nishiyama Y. (2024). Production of extracellular superoxide contributes to photosynthesis via elimination of reducing power and regeneration of NADP(+) in the red-tide-forming raphidophyte *Chattonella marina* complex. Harmful Algae.

[67] Marshall J.-A., Hovenden M., Oda T., Hallegraeff G.M. (2002). Photosynthesis does influence superoxide production in the ichthyotoxic alga *Chattonella marina* (Raphidophyceae). J. Plankton Res..

[68] Brand M.D. (2016). Mitochondrial generation of superoxide and hydrogen peroxide as the source of mitochondrial redox signaling. Free Radic. Biol. Med..

[69] Fu M., Koulman A., van Rijssel M., Lützen A., de Boer M.K., Tyl M.R., Liebezeit G. (2004). Chemical characterisation of three haemolytic compounds from the microalgal species *Fibrocapsa japonica* (Raphidophyceae). Toxicon.

[70] Juergens M.T., Deshpande R.R., Lucker B.F., Park J.J., Wang H., Gargouri M., Holguin F.O., Disbrow B., Schaub T., Skepper J.N. (2015). The regulation of photosynthetic structure and function during nitrogen deprivation in *Chlamydomonas reinhardtii*. Plant Physiol..

[71] Takahashi S., Murata N. (2006). Glycerate-3-phosphate, produced by CO2 fixation in the Calvin cycle, is critical for the synthesis of the D1 protein of photosystem II. Biochim. Biophys. Acta.

[72] Li G., Campbell D.A. (2017). Interactive effects of nitrogen and light on growth rates and RUBISCO content of small and large centric diatoms. Photosynth. Res..

[73] Sato Y., Oda T., Muramatsu T., Matsuyama Y., Honjo T. (2002). Photosensitizing hemolytic toxin in *Heterocapsa circularisquama*, a newly identified harmful red tide dinoflagellate. Aquat. Toxicol..

[74] Afonso S.G., Enríquez de Salamanca R., Batlle A.M. (1999). The photodynamic and non-photodynamic actions of porphyrins. Braz. J. Med. Biol. Res..

[75] Kato H., Komagoe K., Inoue T., Masuda K., Katsu T. (2018). Structure–activity relationship of porphyrin-induced photoinactivation with membrane function in bacteria and erythrocytes. Photochem. Photobiol. Sci..

[76] Basti L., Nagai K., Go J., Okano S., Oda T., Tanaka Y., Nagai S. (2016). Lethal effects of ichthyotoxic raphidophytes, *Chattonella marina*, *C. antiqua*, and *Heterosigma akashiwo*, on post-embryonic stages of the Japanese pearl oyster, *Pinctada fucata martensii*. Harmful Algae.

[77] Flood S.L., Burkholder J.M. (2018). *Chattonella subsalsa* (Raphidophyceae) growth and hemolytic activity in response to agriculturally-derived estuarine contaminants. Harmful Algae.

[78] Sauvage J., Wikfors G.H., Li X., Gluis M., Nevejan N., Sabbe K., Joyce A. (2021). Effect of pluronic block polymers and N-acetylcysteine culture media additives on growth rate and fatty acid composition of six marine microalgae species. Appl. Microbiol. Biotechnol..

[79] Flores H.S., Wikfors G., Dam H. (2012). Reactive oxygen species are linked to the toxicity of the dinoflagellate *Alexandrium* spp. to protists. Aquat. Microb. Ecol..

[80] Granéli E., Flynn K., Granéli E., Turner J.T. (2006). Chemical and physical factors influencing toxin content. Ecology of Harmful Algae.

[81] Vanucci S., Pezzolesi L., Pistocchi R., Ciminiello P., Dell’Aversano C., Iacovo E.D., Fattorusso E., Tartaglione L., Guerrini F. (2012). Nitrogen and phosphorus limitation effects on cell growth, biovolume, and toxin production in *Ostreopsis* cf. *ovata*. Harmful Algae.

[82] Xu Z. (2023). Response of Hemolytic Activity in Relation to Photosynthetic System of Chattonella antiqua.

[83] Guillard R.R., Ryther J.H. (1962). Studies of marine planktonic diatoms. I. *Thalassiosira pseudonana* (*Cyclotella nana*) (Hustedt), and *Detonula confervacea* (Cleve) Gran. Can. J. Microbiol..

[84] Williams O.J., Beckett R.E., Maxwell D.L. (2016). Marine phytoplankton preservation with Lugol’s: A comparison of solutions. J. Appl. Phycol..

[85] Stein J.R. (1973). Handbook of Phycological Methods: Culture Methods and Growth Measurements.

[86] Ling C., Trick C.G. (2010). Expression and standardized measurement of hemolytic activity in *Heterosigma akashiwo*. Harmful Algae.

[87] Song X.R., Xu Z.Y., Zhang W.G., Tong M.M. (2023). Regulation of photosynthetic and hemolytic activity of *Phaeocystis globosa* under different light spectra. New Phytol..

[88] Knauert S., Knauer K. (2008). The Role of Reactive Oxygen Species in Copper Toxicity to Two Freshwater Green Algae. J. Phycol..

