# Supplementary material for: Response of Hemolytic and Photosynthetic Activity of Chattonella marina Complex Under Variable N:P Stoichiometry
Source: Toxins (Basel). 2026 May 9;18(5):226. doi: 10.3390/toxins18050226 (PMC13211709; doi:10.3390/toxins18050226)
Supplement: Supplementary file 1 [file toxins-18-00226-s001.zip › toxins-4286423-supplementary.pdf]

Supplementary Material

Response of Hemolytic and photosynthetic activity of *Chattonella marina* complex under variable stoichiometry

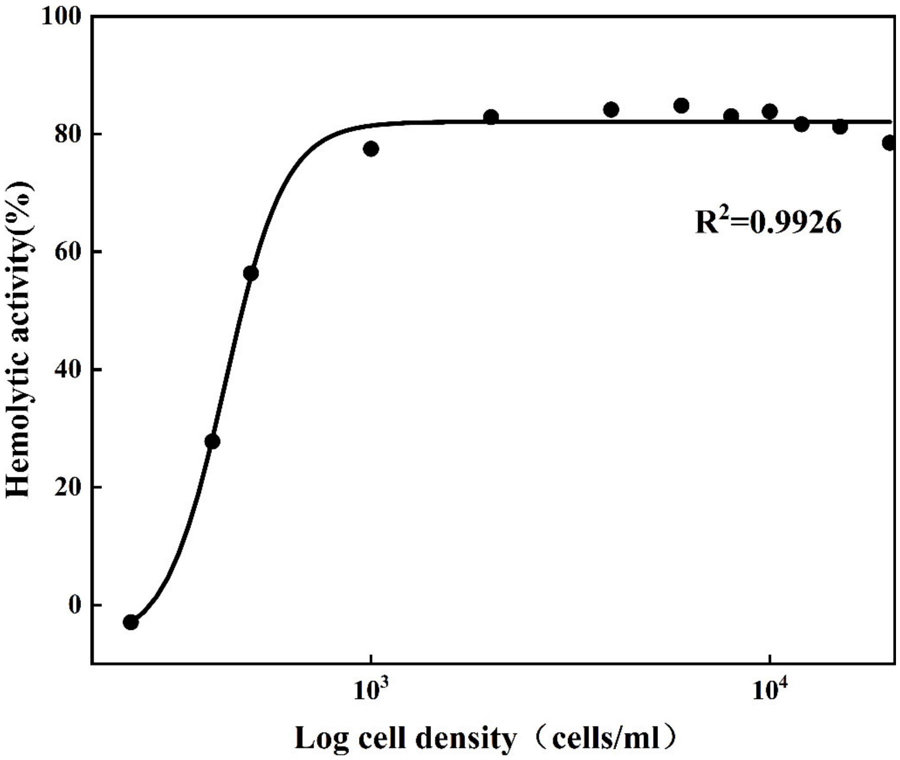

Figure S1 The half-effective concentration (EC_{50}) of *C. marina*

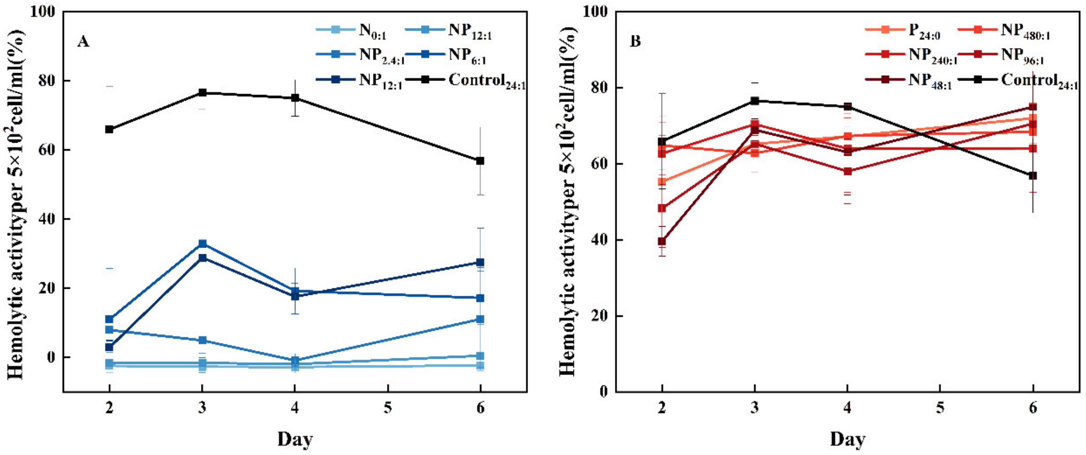

Figure S2 Hemolytic activity of *C. marina* under different stoichiometry conditions.

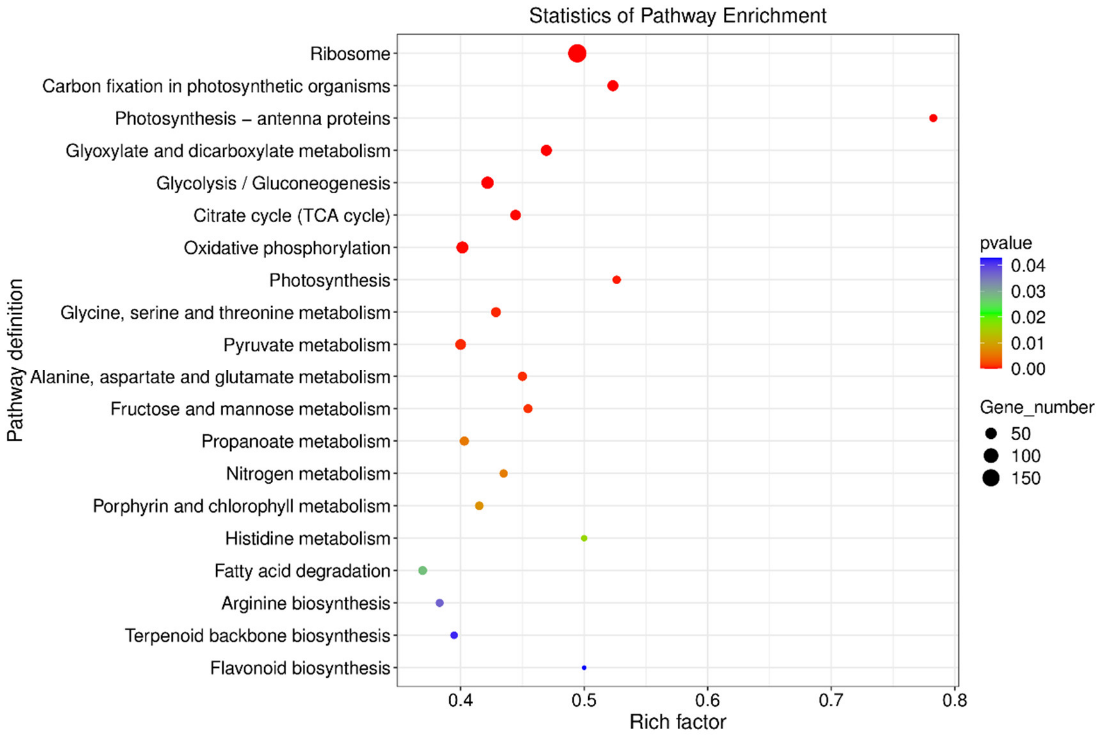

Figure S3 KEGG enrichment analysis of metabolic pathways for differentially expressed genes in *C. marina* under N-limited ($N_{0:1}$) and NP-sufficient ($Control_{24:1}$) conditions.

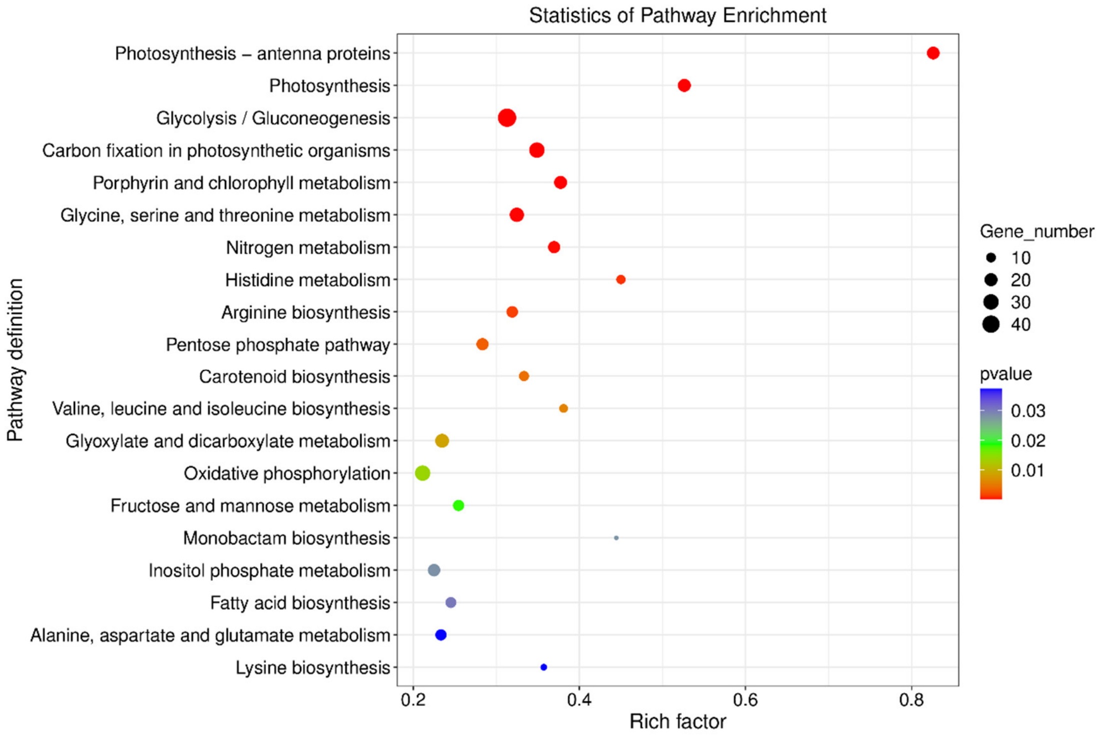

Figure S4 KEGG enrichment analysis of metabolic pathways for differentially expressed genes in *C. marina* under P-limited ($P_{24:0}$) and NP-sufficient ($Control_{24:1}$) conditions.

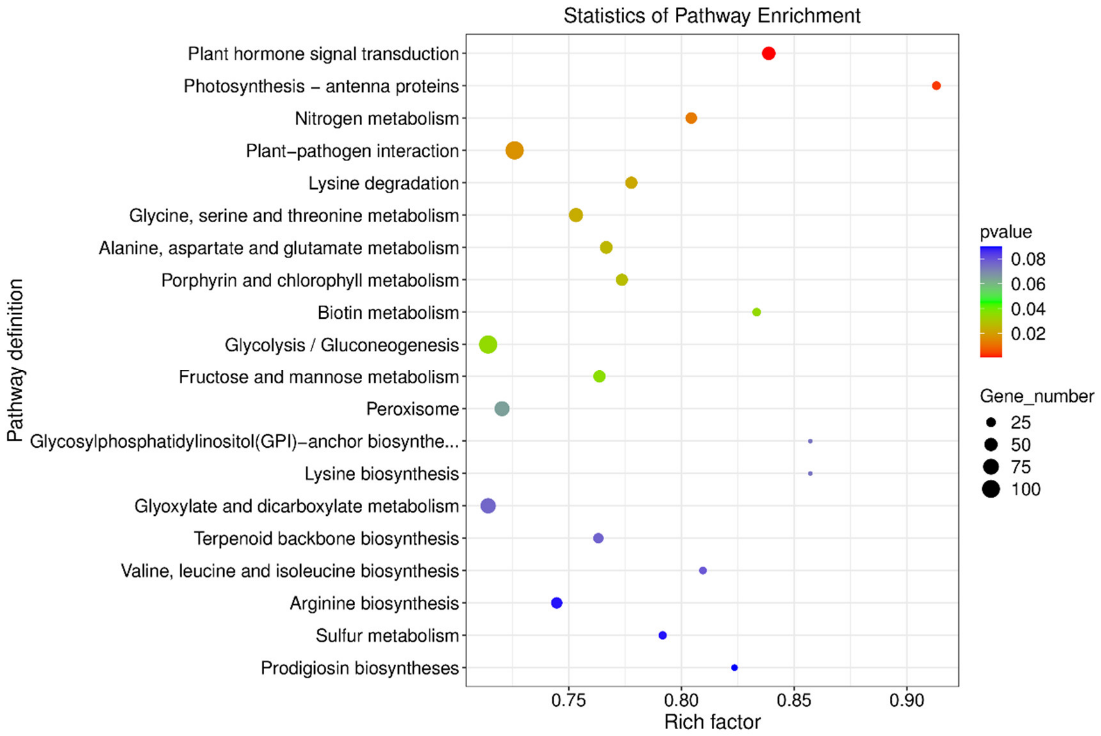

Figure S5 KEGG enrichment analysis of metabolic pathways for differentially expressed genes in *C. marina* under N-limited ($N_{0:1}$), P-limited ($P_{24:0}$) and NP-sufficient ($Control_{24:1}$) conditions.
